# Sex differences in the percentage of IRF5 positive B cells are associated with higher production of TNF-α in women in response to TLR9 in humans

**DOI:** 10.1186/s13293-023-00495-x

**Published:** 2023-02-22

**Authors:** Claudia Beisel, Ana Jordan-Paiz, Sandra Köllmann, Annika Elise Ahrenstorf, Benedetta Padoan, Tanja Barkhausen, Marylyn M. Addo, Marcus Altfeld

**Affiliations:** 1 I. Department of Medicine, University Medical Center Hamburg-Eppendorf, 20251 Hamburg, Germany; 2 German Center for Infection Research (DZIF), University Medical Center Hamburg-Eppendorf, Lübeck-Borstel-Riems, Hamburg, Germany; 3 Research Department Virus Immunology, Leibniz Institute of Virology, 20251 Hamburg, Germany; 4 Department of Internal Medicine IV, Gastroenterology and Infectious Diseases, University Hospital Heidelberg, Im Neuenheimer Feld 410, 69120 Heidelberg, Germany

**Keywords:** B cells, Interferon regulatory factor 5, Sex differences, Toll-like receptor 7/9, Tumor necrosis factor-α

## Abstract

**Background:**

The clinical course and outcome of many diseases differ between women and men, with women experiencing a higher prevalence and more severe pathogenesis of autoimmune diseases. The precise mechanisms underlying these sex differences still remain to be fully understood. IRF5 is a master transcription factor that regulates TLR/MyD88-mediated responses to pathogen-associated molecular patterns (PAMPS) in DCs and B cells. B cells are central effector cells involved in autoimmune diseases via the production of antibodies and pro-inflammatory cytokines as well as mediating T cell help. Dysregulation of IRF5 expression has been reported in autoimmune diseases, including systemic lupus erythematosus, primary Sjögren syndrome, and rheumatoid arthritis.

**Methods:**

In the current study, we analyzed whether the percentage of IRF5 positive B cells differs between women and men and assessed the resulting consequences for the production of inflammatory cytokines after TLR7- or TLR9 stimulation.

**Results:**

The percentage of IRF5 positive B cells was significantly higher in B cells of women compared to men in both unstimulated and TLR7- or TLR9-stimulated B cells. B cells of women produced higher levels of TNF-α in response to TLR9 stimulation.

**Conclusions:**

Taken together, our data contribute to the understanding of sex differences in immune responses and may identify IRF5 as a potential therapeutic target to reduce harmful B cell-mediated immune responses in women.

**Supplementary Information:**

The online version contains supplementary material available at 10.1186/s13293-023-00495-x.

## Highlights

- Women showed higher percentage of IRF5 positive B cells compared to men.
- After stimulation with a TLR7 or TLR9 agonist, women showed higher percentages of IRF5 positive B cells compared with those of men.
- B cells of women produced higher levels of TNF-α in response to TLR9 stimulation.
- IRF5 represents a potential therapeutic target to reduce harmful B cell-mediated immune responses in women.

**Supplementary Information:**

The online version contains supplementary material available at 10.1186/s13293-023-00495-x.

## Background

Differences in immune responses between women and men have been known for a long time but still remain understudied in basic science and clinical studies [1–5]. These differences apply to infections caused by viruses, bacteria, and parasites, including pathogens highly relevant to human health, such as *Human Immunodeficiency Virus (HIV), Mycobacteria tuberculosis, Hepacivirus B,* and* C*, and *Plasmodium falciparum* [6]. In most cases, women show more effective humoral and cell-mediated immune responses to pathogens compared with men [4, 5]. Stronger immune responses in women have also been observed for a variety of vaccine candidates, including vaccines against yellow fever, rubella, measles, mumps, and influenza [5]. Apart from a few exceptions, these robust immune responses in women lead to a higher incidence of immune-related pathology and autoimmunity in women [6–8]. The precise pathways underlying these sex differences in immunity are incompletely understood.

B cells are central to the development of many autoimmune diseases [9–12]. B cells contribute to autoimmunity due to their ability to present antigens to autoreactive T cells, activate inflammatory responses by the production of cytokines and, secretion of autoantibodies [13]. The effectiveness of anti-B cell therapies in the treatment of autoimmune diseases further demonstrates their pathogenic role [11, 14]. B cells can be activated by endogenous deoxyribonucleic acid (DNA) or ribonucleic acid (RNA), through a mechanism that depends amongst others on the toll-like receptor (TLR) 7, and/or TLR9. TLR-stimulated B cells produce a wide range of inflammatory cytokines, including type I interferons (IFNs), tumor necrosis factor-α (TNF-α), and Interleukin-6 (IL-6) [15, 16].

Interferon regulatory factor (IRF) 5 is a key transcription factor for the activation of innate immune responses and is known to play an essential role as an early regulator of human B cell activation, resulting in the production of pro-inflammatory cytokines and antibodies [17]. IRF5 is involved in the immune responses by various TLRs, including TLR7 and/or TLR9 [17, 18]. Autoimmune-risk haplotypes exhibit higher IRF5 levels and are associated with increased levels of pro-inflammatory cytokines, suggesting that expression of IRF5 in B cells contributes to the development of autoimmune diseases [19–21]. Polymorphisms in IRF5 have been associated with multiple autoimmune diseases, particularly in systemic lupus erythematosus (SLE), primary Sjögren syndrome, and rheumatoid arthritis, which are characterized by significant sex differences in the disease prevalence [9, 21, 22].

Our group previously demonstrated that sex differences in the expression of IRF5 lead to higher IFN-α production upon TLR7 stimulation in plasmacytoid dendritic cells (pDCs) of women [23]. Whether sex differences in IRF5 are also present in B cells and might drive differences in the production of inflammatory cytokines and (auto-)antibodies remains unclear. In consequence, sex differences in IRF5 may contribute to stronger immune responses in infectious and immune-mediated diseases as well as in response to vaccinations in women. Here, we describe sex differences in the percentage of IRF5 positive B cells that were associated with altered production of TNF-α upon TLR7/9 stimulation, suggesting that IRF5 might contribute to enhanced immune responses in women.

## Methods

### Study subjects

Healthy participants were recruited at the University Hospital Hamburg Eppendorf. The study was approved by the ethical commission of Ärztekammer Hamburg (PV4780). Prior to study enrollment, each participant gave informed consent.

### Sample processing, isolation of B cells, and stimulation

EDTA–blood was processed within 2 h after venipuncture to prevent loss of responsiveness to stimulation [24]. PBMCs and isolated B cells were maintained in RPMI1640 supplemented with 10% heat-inactivated FBS. B cells were enriched and purified using StemCell EasySep Human B Cell Enrichment Kit (Cat#: 19054). Isolated cells were stimulated for 20 h at 37 °C in the following conditions: 1 μg/mL of TLR7 ligand CL097 (Invivogen, San Diego, CA, US), 500 μM of TLR9 ligand CpG ODN2216 (Invivogen, San Diego, CA, USA), or left unstimulated. The supernatant was collected and frozen at − 80 °C until the LUMINEX assay was performed.

### Staining of IRF5 protein and flow cytometry analysis

PBMCs were stained for surface markers and intracellular IRF5. Cells were fixed using 1% paraformaldehyde and subsequently permeabilized with 0.1% Triton X-100. Fixed cells were first incubated with anti-IRF5 antibody (Cell Signaling Technology, Cat#: 76983, clone E7F9W) for 20 min and then a secondary anti-rabbit-IgG AlexaFluor488 antibody (Cell Signaling Technology, Cat#: 4412) for 15 min. In the next step, the cells were washed and stained using fluorochrome-conjugated surface antibodies anti-CD3 BUV737 (BD Bioscience, Cat#: 564307, clone UCHT1), anti-IgM BV421 (Biolegend, Cat#: 314516, clone MHM-88), anti-IgD Per-CP-cy5.5 (Biolegend, Cat#: 348208, clone IA6-2), anti-CD20 PE-Cy7 (Biolegend, Cat#: 302312, clone 2H7) (see Additional file 1: Table S1). Samples were acquired within 6 h on a BD LSRFortessa II (BD Biosciences). Cells were analyzed using FlowJo 10.7 software (BD Biosciences) with the exclusion of doublet cells. B cells were identified as CD3negative CD20 positive cells. The functionality of surface antibodies was compared on fixed and unfixed cells, showing a reliable result for all antibodies used (see Additional file 1: Fig. S1).

### Real-time PCR

RNA was purified using TRIzol (Life Technologies, Carlsbad, CA; US)/chloroform ultrapure (Applichem, Darmstadt, Germany) and RNeasy Mini kit (Qiagen, Hilden, Germany) according to the manufacturer’s instructions. cDNA was prepared using qScriptTM cDNA SuperMix (Quanta BioSciences, Gaithersburg, Maryland, US) according to the manufacturer’s instructions. PCR was performed using Quanti fast SYBR green (Qiagen). Samples were run in duplicates on the LightCycler® 480 System (Roche, Basel, Switzerland) and values were calculated in relation to housekeeping gene GAPDH.

### LUMINEX assay

To assess cytokine production by isolated B cells, we performed a LUMINEX assay of cell supernatant collected after 20 h of stimulation with TLR9 ligand CpG ODN2216 and after 20 h of unstimulated condition. For the measurements, we used the MILLIPLEX MAP Kit (Merck KGAA, Darmstadt, Germany). Samples were run in triplicates. The protocol was performed according to the manufacturer’s instructions in a 96-well plate with 25 µl of supernatant for cellular protein quantification. Protein quantification was done using the Bio-Plex-System 200 (Bio-Rad Laboratories GmbH, Feldenkirchen, Germany) and the Bio-Plex Manager™ 4.1.1 software (Bio-Rad Laboratories GmbH, Feldenkirchen, Germany), measured in mean fluorescence intensity (MFI). The MFI values were normalized using protein lysate concentrations measured according to the Bradford method.

### Statistical analyses

GraphPad Prism 5 (GraphPad Software) was used to analyze the data. Two-tailed *t* tests were used to determine statistical significance between the groups. Linear regression was calculated with Pearson's correlation coefficient. A *p* value < 0.05 was considered significant.

## Results

### Demographics

Between January 2018 and August 2020, a total of 22 healthy individuals were enrolled in this study. Sex ratios were balanced in the study cohort (women *n* = 11, men *n* = 11). At the time of study inclusion, the age of the study subjects ranged from 24 to 60 years. The mean age of the cohort was 31.2 years. The mean age of the male individuals was 29 years (range 24–33 years). The ages of the female study individuals varied from 27 to 60 years (mean age 33,4 years). With the exception of one, all female donors were premenopausal women having a regular menstrual cycle and were age-matched to their male controls. All donors showed no sign of acute or chronic infection and did not suffer from any autoimmune-related diseases.

### Higher percentages of IRF5 positive B cells in women compared with men ex vivo

**Fig. 1:**
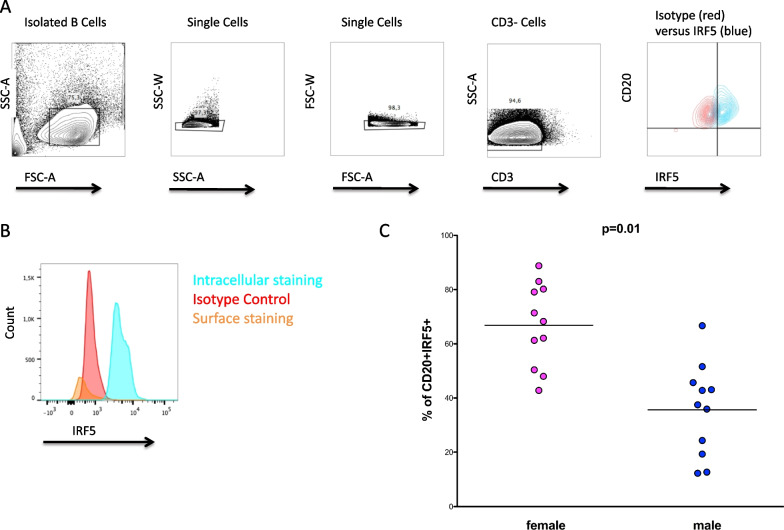
Sex differences in the percentages of IRF5 positive B cells. **A** Flow cytometric plots showing the applied gating strategy for isolated B cells (CD3–CD20 + cells). FACS plots of a representative donor are shown (out of 22 experiments). B cells were purified with Human B Cell Enrichment Cocktail. The percentages of IRF5 positive B cells were analyzed ex vivo by flow cytometry. The first gate was set on physical parameters, then on SSC-W versus SSC-A and FSC-W versus FSC-A to eliminate doublets, then on CD3 negative events to exclude remaining CD3 positive cells. IRF5 levels (in blue) were determined by the use of an isotype control (in red) in B cells from women or age-matched men. **B** To confirm antibody specificity, we performed surface staining to detect IRF5 on the cell surface of CD20 + B cells. IRF5 staining was performed by surface staining (orange histogram) and after permeabilized in 0.01% Triton-X-100 for intracellular staining (blue histogram) or isotype control (red histogram). Surface staining of IRF5 served as a negative control as IRF5 is located in the cytoplasm and intranuclearly [25]. No IRF5 positive B cells were detected after surface staining alone. **C** Percentages of IRF5 positive B cells. Women (pink dots) showed significantly higher percentage of IRF5 positive B cells than men (blue dots) in unstimulated conditions (*p* = 0.01; two-tailed *t* test; women *n* = 11, men *n* = 11)

IRF5 expression is mainly restricted to dendritic cells (DC) and B cells [25–27]. To determine whether B cells from healthy women and men differ in the percentages of IRF5 positive cells, we isolated B cells from fresh peripheral blood mononuclear cells (PBMC) by negative selection in a group of 22 healthy individuals (11 women and 11 men) and analyzed the percentages of IRF5 positive B cells using flow cytometry. The applied gating strategy to identify IRF5 positive CD20 + B cells is shown in Fig. 1A. In a quiescent cell, IRF5 protein resides in the cytoplasm. Upon stimulation, IRF5 becomes activated via posttranslational modification, which results in nuclear translocation [25]. To confirm antibody specificity, we identified IRF5 + CD3 + T cells and compared the percentages of IRF5 positive cells after surface staining and intracellular staining. In line with the literature [28], antibody specificity was considered to be reliable as no IRF5 + CD3 + T cells could be identified (see Additional file 1: Fig. S2). After surface staining alone, no IRF5 positive B cells were detected (see Fig. 1B). Overall, we observed a significantly higher percentage of IRF5 positive B cells derived from women compared with B cells derived from men (*p* = 0.01) (see Fig. 1C), while the mean fluorescent intensity (MFI) of IRF5 in B cells from women was only slightly increased compared to men (mean of 11,708 versus 9994; *p* = 0.23; data not shown). Furthermore, no differences in IRF5 mRNA expression levels were observed in isolated B cells using qRT-PCR (*p* = 0.78) (see Additional file 1: Fig. S3), indicating that posttranscriptional effects might contribute to the observed sex differences in the percentages of IRF5 positive B cells.

### Higher percentages of IRF5 positive B cells in women compared with men following TLR-mediated stimulation (TLR7 or TLR9) ex vivo

**Fig. 2:**
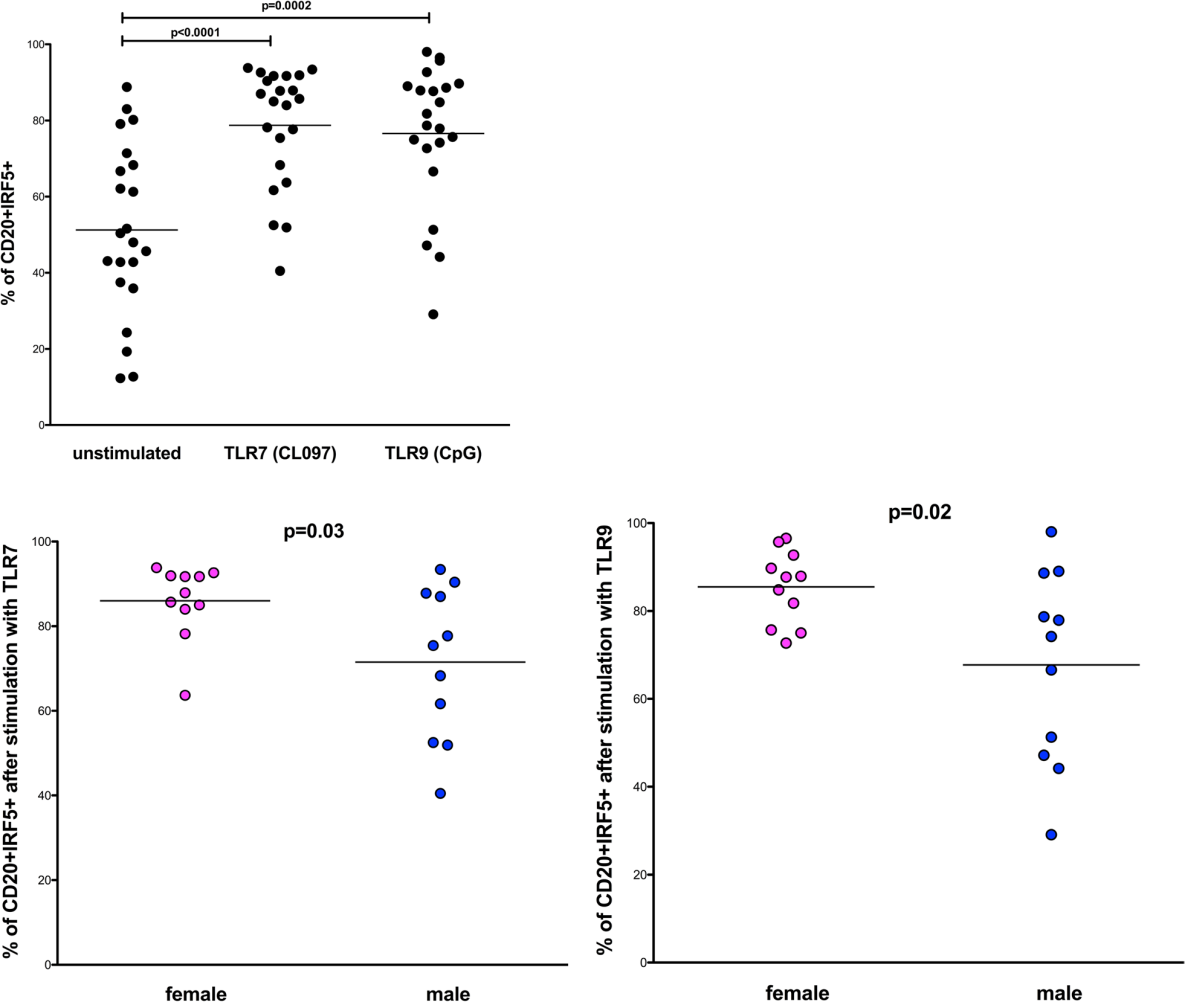
Sex differences in the percentage of IRF5 positive B cells ex vivo following TLR7 and TLR9 stimulation. Percentages of IRF5 positive B cells were defined by Flow Cytometry with the aid of an isotype control. Upper row: percentages of IRF5 + CD20 + B cells are shown in an unstimulated condition and after 20 h of stimulation with a TLR7 agonist (CL097) and a TLR9 agonist (CpG (ODN 2216)). Percentages of IRF5 positive B cells increased significantly after TLR7 stimulation (*p* < 0.0001) and after TLR9 stimulation (*p* = 0.0002; two-tailed *t* test; women *n* = 11, men *n* = 11). Lower row left: women (pink dots) showed significantly higher percentages of IRF5 positive B cells than men (blue dots) after 20 h of TLR7 stimulation (*p* = 0.03; two-tailed *t* test; women *n* = 11, men *n* = 11). Lower row right: women (pink dots) showed significantly higher percentages of IRF5 positive B cells than men (blue dots) after 20 h of TLR9 stimulation (*p* = 0.02; two-tailed *t* test; women *n* = 11, men *n* = 11)

IRF5 is a central transcription factor activated upon TLR7- and/or TLR9-stimulation that induces the production of inflammatory cytokines [29, 30]. To determine whether TLR-stimulation induces upregulation of IRF5 in B cells and whether the extent of IRF5 induction differs between women and men, we analyzed percentages of IRF5 positive B cells after 20 h of stimulation with the TLR7 agonist CL097 or the TLR9 agonist CpC (ODN 2216) using flow cytometry. Percentages of IRF5 positive B cells increased significantly after both TLR7- and TLR9-stimulation compared with unstimulated B cells (see Fig. 2 upper row) (*p* < 0.0001 and *p* = 0.0002). Furthermore, women showed significantly higher percentages of IRF5 positive B cells compared with those of men after stimulation with both TLR7 and TLR9 agonists (see Fig. 2, lower row) (*p* = 0.03 and *p* = 0.02). While the percentages of IRF5 positive B cells significantly differed between the sexes, no differences were observed in the MFI of IRF5 upon TLR7 stimulation (mean of 19,607 versus 18,761; *p* = 0.76; data not shown) or TLR9-stimulation (mean of 18,794 versus 17,805; *p* = 0.76; data not shown). These data demonstrate that the percentages of IRF5 positive B cells increase upon stimulation of the TLR7 or 9 signaling pathway and that as observed for unstimulated B cells, women show higher percentages of IRF5 positive B cells in response to TLR7 or TLR 9 stimulation. Taken together, these sex differences in IRF5 suggest a regulatory role for TLR7/9-dependent B cell responses.

### Higher production of tumor necrosis factor-α (TNF-α) by isolated B cells following TLR9-stimulation in women

**Fig. 3:**
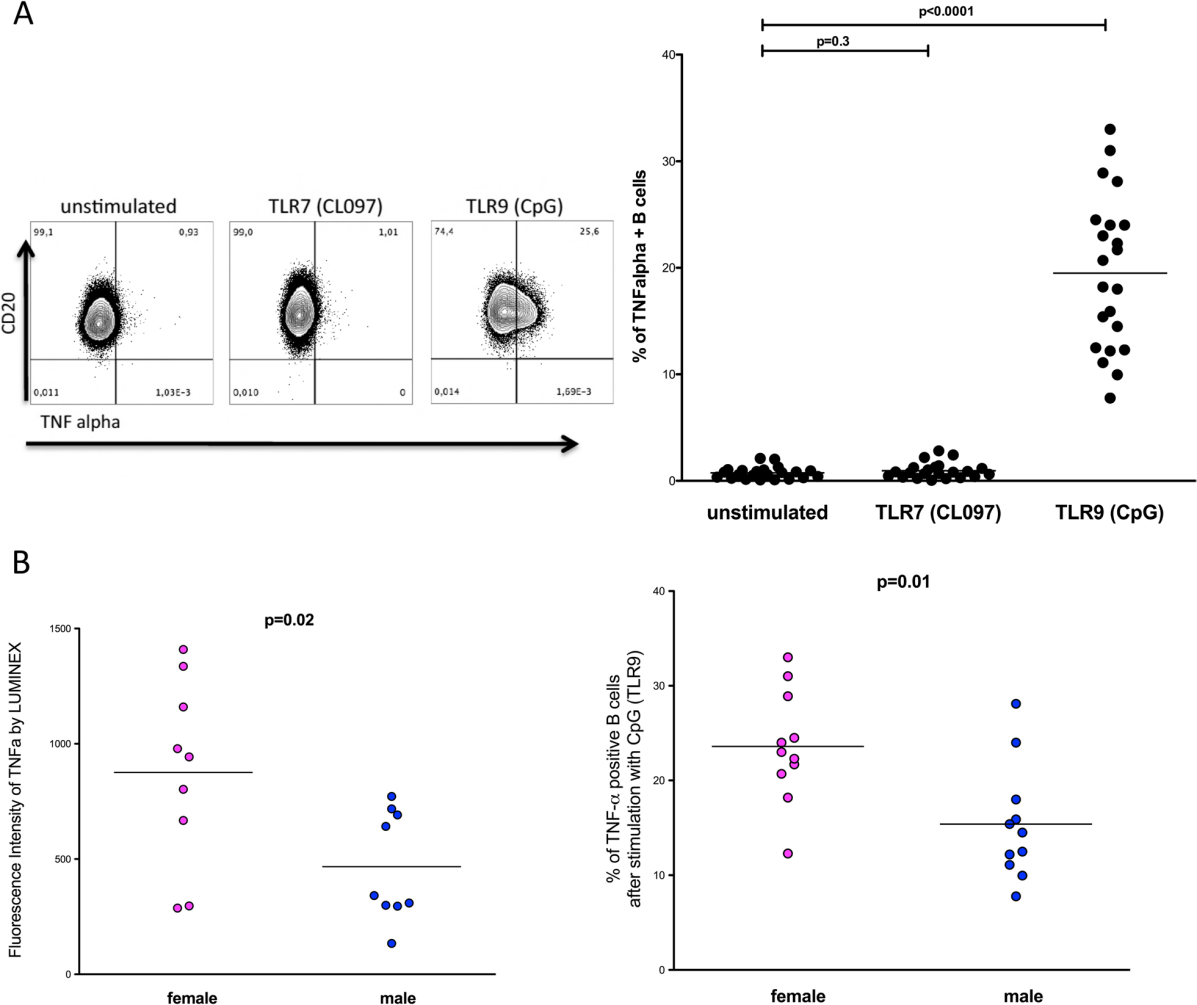
Sex differences in the production of TNF-α by B cells following stimulation with a TLR7 and TLR9 agonist. **A** A representative flow cytometry analysis to identify TNF-α positive B cells in an unstimulated condition (left), after 20 h of stimulation with the TLR7 (CL097) agonist (middle) and the TLR9 agonist (CpG (ODN 2216)) (right) is shown. The assessment of the percentages of TNF-α positive B cells is shown in an unstimulated condition, after stimulation with the TLR7 agonist (CL097) or the TLR9 agonist (CpG (ODN 2216)) in a group of 22 healthy individuals. No increase of TNF-α positive B cells was detected upon TLR7 stimulation. The percentage of TNF-α positive B cells increased significantly upon stimulation with the TLR9 agonist compared with unstimulated B cells (*p* < 0.0001; two-tailed *t* test; women *n* = 11, men *n* = 11). **B** Left figure: levels of TNF-α were measured in the supernatant of isolated B cells after stimulation with a TLR9 agonist CpG (ODN 2216) using Luminex multiplex technology. The fluorescence intensity of TNF-α is illustrated in the dot plots in women (pink dots) and men (blue dots). B cells of women produced significantly higher levels of TNF-α than B cells of men after stimulation of TLR9 (*p* = 0.02; two-tailed *t* test; women *n* = 9, men *n* = 9). Right figure: TNF-α positive B cells were determined ex vivo from female or age-matched male donors using FlowJo software. Women (pink dots) showed a significantly higher percentage of TNF-α producing B cells in response to stimulation with CpG (ODN 2216) compared with men (blue dots) (*p* = 0.01; two-tailed *t* test; women *n* = 11, men *n* = 11)

TLR-stimulated B cells produce a wide range of inflammatory cytokines, including TNF-α, and IRF5 has been described as a master regulator of inflammatory cytokines following TLR stimulation [17]. To determine the consequences of sex difference in IRF5 positive B cells for the production of TNF-α, we quantified TNF-α-producing B cells by flow cytometry following stimulation with a TLR7 (CL097) or TLR9 agonist (CpG (ODN 2216)). While TLR7-stimulation did not induce TNF-α production in B cells (*p* = 0.3), TLR9-stimulation resulted in a significant increase in TNF-α-producing B cells (see Fig. 3A) (*p* < 0.0001). Furthermore, a significantly higher percentage of TNF-α-producing B cells was observed in women compared to men in response to TLR9-stimulation (*p* = 0.01) (see Fig. 3B). To confirm that a higher percentage of TNF-α-producing B cells quantified by intracellular cytokine staining using flow cytometry corresponded to higher levels of secreted TNF-α, we quantified the amount of TNF-α in the supernatant of B cells following TLR9 stimulation (CpG (ODN 2216)) using a LUMINEX assay. Consistent with the flow cytometry data, B cells of women produced significantly more TNF-α in response to TLR9 stimulation compared with B cells of men (see Fig. 3B; *p* = 0.02).

### Higher percentages of IRF5 positive CD20 + IgD–IgM + immature B cells but not IRF5 positive CD20 + IgD + IgM + mature naïve B cells of women

**Fig. 4:**
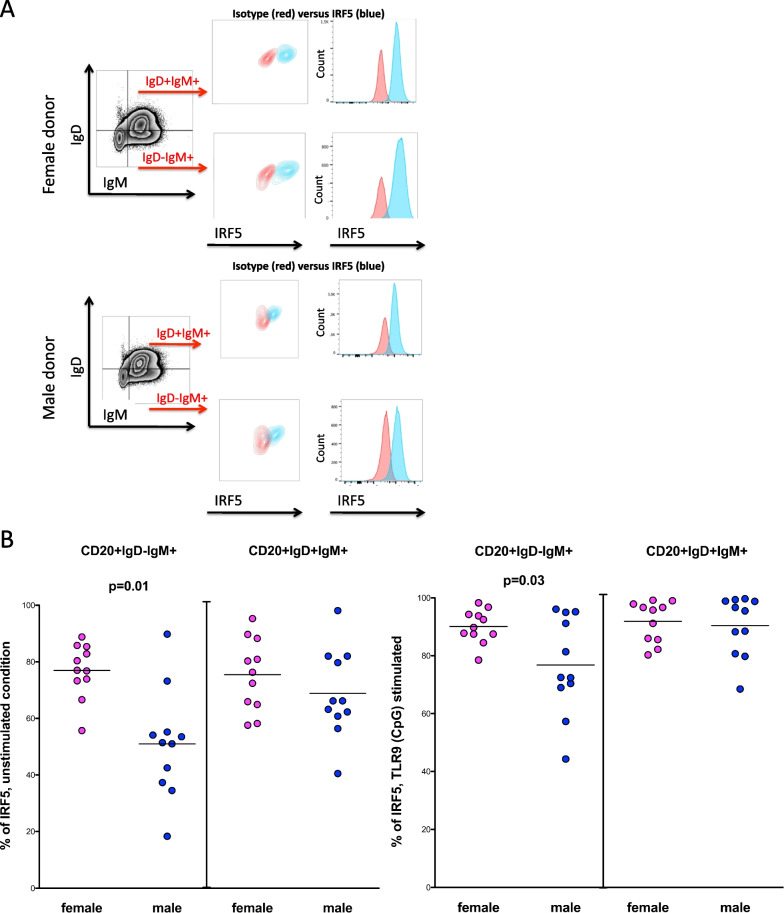
Sex differences in the percentages of IRF5 positive CD20 + IgD–IgM + immature B cells and but not of IRF5 positive CD20 + IgD + IgM + mature naïve B cells. **A** Flow cytometric plots showing the applied gating strategy for IgD–IgM + , and IgD + IgM + B cells in a representative woman and man (out of 22 experiments). B cells were purified with Human B Cell Enrichment Cocktail. Gates distinguishing IgD + and IgD-cells were set plotting IgD against the side scatter (SSC-A) on B cells, as this enabled a clear distinction of IgD positive and negative B cell populations. Percentages of IRF5 positive cells were analyzed ex vivo by flow cytometry. IRF5 positive cells (in blue) were determined by the use of an isotype control (in red). **B** Left: under unstimulated conditions, women showed significantly higher percentages of IRF5 positive CD20 + IgD–IgM + immature B cells than men (*p* = 0.01; two-tailed *t* test; women *n* = 11, men *n* = 11). The percentage of IRF5 positive CD20 + IgD + IgM + mature naïve B cells in women was only slightly higher than the percentage of IRF5 positive CD20 + IgD + IgM + mature naïve B cells in men. Right: after 20 h of stimulation with the TLR9 agonist CpG (ODN 2216), women still showed a significantly higher percentages of IRF5 positive CD20 + IgD–IgM + immature B cells compared to those of men (*p* = 0.03; two-tailed *t* test; women *n* = 11, men *n* = 11). No significant differences in the percentages of IRF5 positive CD20 + IgD + IgM + mature naïve B cells were observed between the sexes. Pink dots indicate women, blue dots indicate men donors

Next, we wanted to assess whether the observed sex differences in the percentage of IRF5 positive B cells might play a role in the early humoral immune response when naïve B cells expressing IgM and IgD represent a critical B cell subset [31]. We, therefore, analyzed the percentage of IRF5 positive CD20 + IgD–IgM + immature B cells and IRF5 positive CD20 + IgD + IgM + mature naïve B cells (see Fig. 4A). Under unstimulated condition, women showed a significantly higher percentage of IRF5 positive CD20 + IgD–IgM + immature B cells than men (mean of 76.9% versus 59.9%; *p* = 0.01; two-tailed *t* test; women *n* = 11, men *n* = 11) (Fig. 4B). In contrast, the percentage of IRF5 positive CD20 + IgD + IgM + mature naïve B cells of women differed not significantly between the sexes (mean of 75.4% versus 68.8%; *p* = 0.3). After stimulation with the TLR9 agonist CpG (ODN 2216), there were again no significant differences in the percentages of IRF5 positive CD20 + IgD + IgM + mature naïve B cells between women and men (mean of 91.8% versus 90.4%; *p* = 0.7), whereas women showed significantly higher percentages of IRF5 positive CD20 + IgD–IgM + immature B cells to those of men (mean of 90.12% versus 76.8%; *p* = 0.03) (see Fig. 4B). Taken together, sex differences were detected in the percentage of IRF5 positive CD20 + IgD–IgM + immature B cells, but not in IRF5 positive CD20 + IgD + IgM + mature naïve B cells, suggesting that higher percentages of IRF5 positive CD20 + IgD–IgM + immature B cells exist in an early stage of B cell development in women.

## Discussion

Despite important differences between women and men in infectious and autoimmune diseases, as well as in the immune responses against several vaccinations [6], the mechanisms underlying these sex differences remain incompletely understood. Polymorphisms in the IRF5 gene have been associated with an increased risk of numerous autoimmune diseases, including SLE, primary Sjögren syndrome, and rheumatoid arthritis [32, 33]. Recent data, furthermore, described that inhibition of IRF5 hyperactivation protected from lupus onset and diseases severity [34]. Moreover, Ban et al. showed that inhibition of IRF5 after disease onset suppressed disease progression and was effective for maintenance of remission in mice. Overall, these studies underline that IRF5 represents a valuable therapeutic target to treat autoimmune and inflammatory diseases [34, 35].

Although it has become clear that sex importantly influences immune responses, most studies did not examine whether sex differences exist in IRF5 polymorphisms and if they alter the production of related cytokines. Recent studies have also elucidated that aging impairs immune responses with an increased risk and severity of infections in the elderly population [36–38]. In general, these studies show that human TLR function is impaired in the context of aging. To rule out age as a confounding factor, all donors of our cohort were age-matched with the exception of one female donor being post-menopausal.

In 2015, Griesbeck et al. described a mechanism by which differences in expression levels of IRF5 in pDCs resulted in a higher percentage of IFN-α-producing pDCs following TLR7-stimulation in women [23]. Moreover, IRF5 is known for its intrinsic role in the activation, proliferation, and differentiation of B cells [39]. We, therefore, aimed to examine whether percentages of IRF5 positive B cells also underlie sex differences and contribute to differences in the production of inflammatory cytokines between women and men. First, we examined percentages of IRF5 positive B cells in unstimulated conditions. Significantly higher percentages of IRF5 positive B cells were found in women compared with men. IRF5 is well-characterized to act downstream of TLR signaling in monocytes, dendritic cells, and B cells [39]. B cells are capable of receptor-mediated responses to foreign antigens. Recognition of nucleic acid (NA) by TLR7 and TLR9 in B cells has been conclusively established [15, 16]. Endogenous NA released from damaged or dead cells can also be immunogenic and can induce harmful activation of B cells, potentially accounting for immune-mediated diseases that are generally more common in women [4]. In the context of HIV-1 infection, it is well-established that the frequency of pDCs producing IFN-α upon TLR7 stimulation is significantly higher in women than in men, correlating with clinical differences in the course of HIV-1 infection [40–42]. To the best of our knowledge, this study addresses for the first-time sex differences in TLR-mediated signaling pathways in B cells. Significantly higher percentages of IRF5 positive B cells derived from women compared with those from men unstimulated and following stimulation with a TLR7 or TLR9 agonist were detected. Immunotherapeutic applications of TLR agonists include not only approaches to enhance immune responses, but also immunosuppressive strategies for treating autoimmune diseases [43, 44]. Although these findings suggest that modulation of the TLR7 and/or 9 signaling pathway may represent a potentially attractive target for the treatment of autoimmune or inflammatory disorders, future studies will be required to replicate these data in respective disease models or clinical cohorts.

We subsequently determined whether these sex differences influence the production of inflammatory cytokines by B cells. In addition to their well-established role in antibody production, B cells may regulate immune responses through their production of cytokines, including TNF-α [45]. Inflammatory cytokines such as TNF-α are expressed at high levels in patients with various autoimmune diseases, where they contribute significantly to chronic inflammation [46]. TNF-α was the first cytokine to be validated as a therapeutic target for rheumatoid arthritis. Until today, the TNF-α inhibitors have revolutionized the treatment of many other immune-mediated diseases, such as inflammatory bowel disease, ankylosing spondylitis, and psoriasis [47]. Previous studies have reported conflicting results regarding sex differences in cytokine levels. These studies varied in cell types and stimulations used, suggesting that the impact of sex on the production of inflammatory cytokines depends on cell type and ligands used [45, 48, 49]. Our study demonstrates that women exhibited a higher number of TNF-α-producing B cells in response to TLR9 stimulation. In line with this, women showed significantly higher levels of TNF-α in the supernatant of TLR9 stimulated B cells compared with men. These findings suggest that sex differences in the percentages of IRF5 positive B cells contribute to higher production of TNF-α in women.

To investigate whether the observed sex differences in CD20 + B cells contributed to an enhanced early B cell response in women, we analyzed the percentages of IRF5 positive CD20 + IgD–IgM + immature B cells and of IRF5 positive CD20 + IgD + IgM + mature naïve B cells. Interestingly, no sex differences were detected in the percentages of IRF5 positive mature naïve B cells, while percentages of IRF5 positive immature B cells of women were significantly higher under unstimulated conditions and upon TLR9 stimulation compared to those of men. In summary, these findings indicate that higher percentages of IRF5 positive immature B cells play a role in the very early stage of B cell development. Whether the observed sex differences in IRF5 contribute to the observed higher prevalence of autoimmune diseases in women needs to be assessed in future studies. A main limitation of this study is, furthermore, its small sample size, which limits the generalizability of the study's findings. Nevertheless, our data underline that sex is a biological variable that should be considered in immunological studies.

### Perspectives and significance

Our data shows that IRF5 can contribute to the mechanisms underlying sex differences in human immune responses in B cells. Taken together, this study demonstrates that the percentages of IRF5 in the B cells of women are higher at baseline and upon stimulation with TLR7 or TLR9 agonists. Furthermore, in response to TLR9 stimulation, isolated B cells of women produced significantly higher levels of TNF-α than those of men. These results indicate IRF5 as a potential target for specific modulation of harmful immune responses—most important in B-cell-triggered autoimmune diseases.

## Supplementary Information

**Additional file 1: Figure S1.** Comparison of antibody functionality on fixed and unfixed cells. To confirm whether all surface antibodies used were applicable on fixed and unfixed cells, we compared two different staining protocols. In the upper row intracellular and surface staining was done before cells were fixed using 1% paraformaldehyde. In the lower row no paraformaldehyde was used, thus staining was performed on unfixed cells. Flow cytometric plots showing the applied gating strategy to identify CD20+IgD–IgM+ immature B Cells and CD20+IgD+IgM+ mature naïve B cells. The first gate was set on physical parameters, then on SSC-W versus SSC-A and FSC-W versus SSC-W to eliminate doublets, then on CD3-events, followed on CD20+ to analyze CD20+ B cells. In the next step, IgD versus IgM on CD20+ B cells is shown to differentiate CD20+IgD–IgM+ immature B Cells and CD20+IgD+IgM+ mature naïve B cells. **Figure S2.** Gating strategy used to identify IRF5+CD3+ T cells to determine antibody specificity. Flow cytometric plots showing the applied gating strategy to identify IRF5+CD3+ T cells. FACS plots of a representative donor are shown. CD3+ T cells were used as a negative control as they are known to exhibit no IRF5. The first gate was set on physical parameters, then on SSC-W versus SSC-A and FSC-W versus SSC-W to eliminate doublets, then on CD3+ events, or on CD3-cells followed on CD20+ to analyze CD20+ B cells. The upper row shows the isotype control for IRF5 on CD3+ T cells. In the middle row, the percentage of IRF5+CD3+ T cells is shown (physiological negative control) (1.1%). The percentage of IRF5+CD20+ B cells is shown in the lower row (29.6%). **Figure S3.** mRNA expression levels of IRF5 in isolated B cells. mRNA expression levels of IRF5 in isolated B cells derived from females and males relative to GAPDH. No sex differences in expression levels of IRF5 mRNA could be detected (p=0.78, two-tailed *t* test; females n=5, males n=4). **Table S1.** Antibodies used in this study.

## Data Availability

Data storage is performed by the Leibniz Institute of Virology. Data are available upon request from the corresponding author and can be shared after confirming that data will be used within the scope of the originally provided informed consent.
