# Supplementary material for: Sex differences in the percentage of IRF5 positive B cells are associated with higher production of TNF-α in women in response to TLR9 in humans
Source: Biol Sex Differ. 2023 Feb 22;14:11. doi: 10.1186/s13293-023-00495-x (PMC9945365; doi:10.1186/s13293-023-00495-x)
Supplement: Supplementary file 1 — Additional file 1: Figure S1. Comparison of antibody functionality on fixed and unfixed cells. To confirm whether all surface antibodies used were applicable on fixed and unfixed cells, we compared two different staining protocols. In the upper row intracellular and surface staining was done before cells were fixed using 1% paraformaldehyde. In the lower row no paraformaldehyde was used, thus staining was performed on unfixed cells. Flow cytometric plots showing the applied gating strategy to identify CD20+IgD–IgM+ immature B Cells and CD20+IgD+IgM+ mature naïve B cells. The first gate was set on physical parameters, then on SSC-W versus SSC-A and FSC-W versus SSC-W to eliminate doublets, then on CD3-events, followed on CD20+ to analyze CD20+ B cells. In the next step, IgD versus IgM on CD20+ B cells is shown to differentiate CD20+IgD–IgM+ immature B Cells and CD20+IgD+IgM+ mature naïve B cells. Figure S2. Gating strategy used to identify IRF5+CD3+ T cells to determine antibody specificity. Flow cytometric plots showing the applied gating strategy to identify IRF5+CD3+ T cells. FACS plots of a representative donor are shown. CD3+ T cells were used as a negative control as they are known to exhibit no IRF5. The first gate was set on physical parameters, then on SSC-W versus SSC-A and FSC-W versus SSC-W to eliminate doublets, then on CD3+ events, or on CD3-cells followed on CD20+ to analyze CD20+ B cells. The upper row shows the isotype control for IRF5 on CD3+ T cells. In the middle row, the percentage of IRF5+CD3+ T cells is shown (physiological negative control) (1.1%). The percentage of IRF5+CD20+ B cells is shown in the lower row (29.6%). Figure S3. mRNA expression levels of IRF5 in isolated B cells. mRNA expression levels of IRF5 in isolated B cells derived from females and males relative to GAPDH. No sex differences in expression levels of IRF5 mRNA could be detected (p=0.78, two-tailed t test; females n=5, males n=4). Table S1. Antibodies used in this study. [file 13293_2023_495_MOESM1_ESM.pdf]

Additional Figures

“Sex differences in the percentage of IRF5 positive B cells are associated with higher production of TNF-α in females in response to TLR9 in humans.“

Beisel et al.

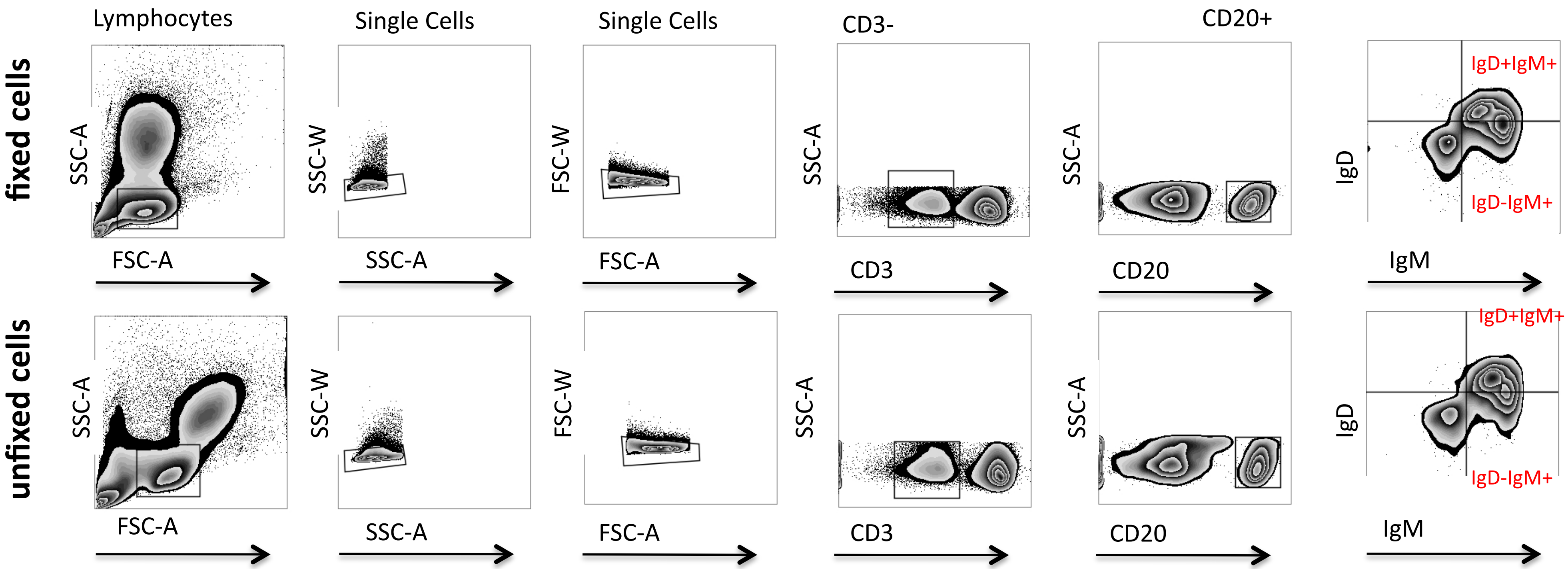

Figure S1: Comparison of antibody functionality on fixed and unfixed cells.
To confirm whether all surface antibodies used were applicable on fixed and unfixed cells, we compared two different staining protocols. In the upper row intracellular and surface staining was done before cells were fixed using 1% paraformaldehyde. In the lower row no paraformaldehyde was used, thus staining was performed on unfixed cells. Flow cytometric plots showing the applied gating strategy to identify CD20+IgD-IgM+ immature B Cells and CD20+IgD+IgM+ mature naïve B cells. The first gate was set on physical parameters, then on SSC-W versus SSC-A and FSC-W versus SSC-W to eliminate doublets, then on CD3- events, followed on CD20+ to analyze CD20+ B cells. In the next step, IgD versus IgM on CD20+ B cells is shown to differentiate CD20+IgD-IgM+ immature B Cells and CD20+IgD+IgM+ mature naïve B cells.

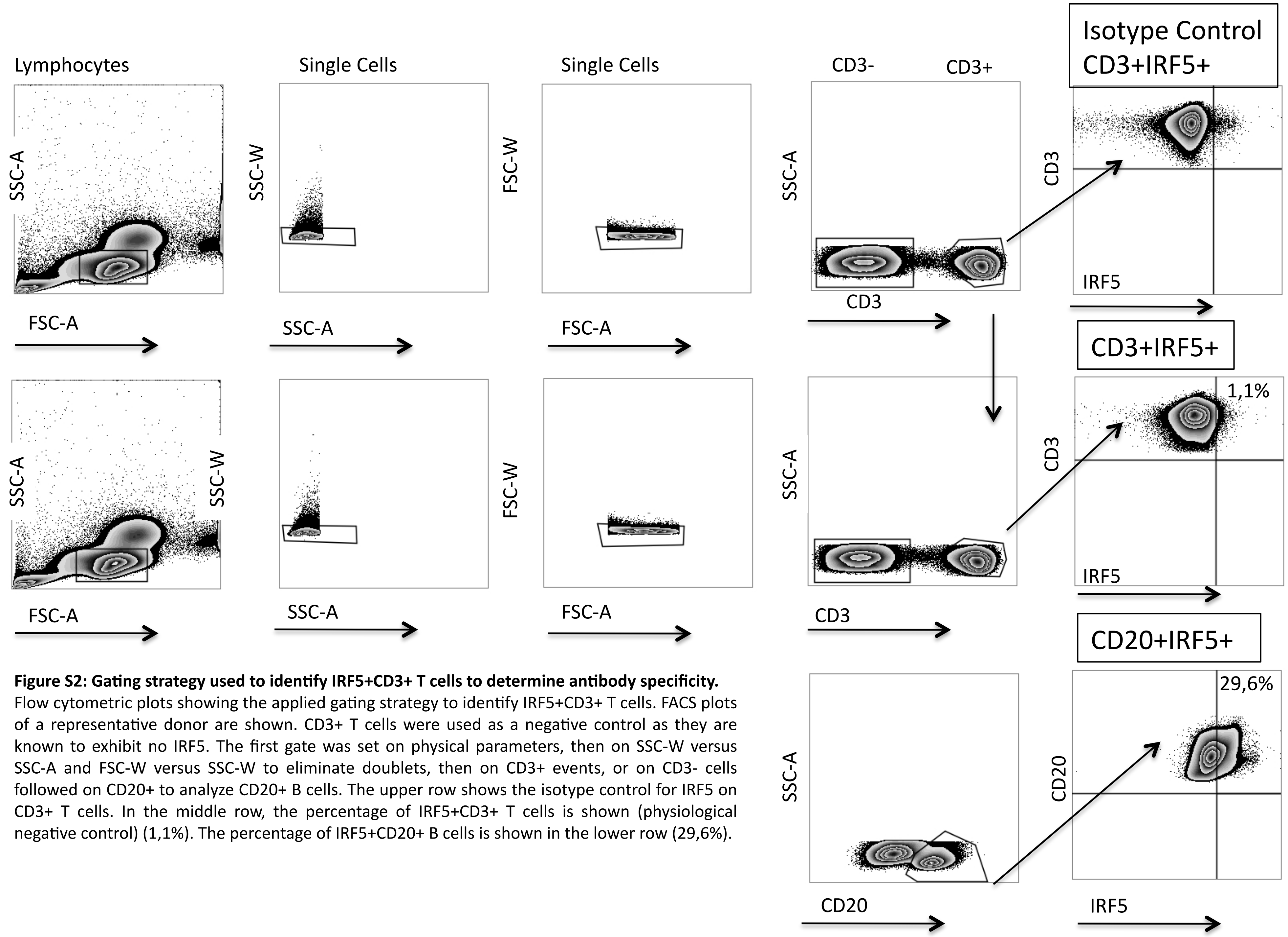

Figure S2: Gating strategy used to identify IRF5+CD3+ T cells to determine antibody specificity. Flow cytometric plots showing the applied gating strategy to identify IRF5+CD3+ T cells. FACS plots of a representative donor are shown. CD3+ T cells were used as a negative control as they are known to exhibit no IRF5. The first gate was set on physical parameters, then on SSC-W versus SSC-A and FSC-W versus SSC-W to eliminate doublets, then on CD3+ events, or on CD3- cells followed on CD20+ to analyze CD20+ B cells. The upper row shows the isotype control for IRF5 on CD3+ T cells. In the middle row, the percentage of IRF5+CD3+ T cells is shown (physiological negative control) (1,1%). The percentage of IRF5+CD20+ B cells is shown in the lower row (29,6%).

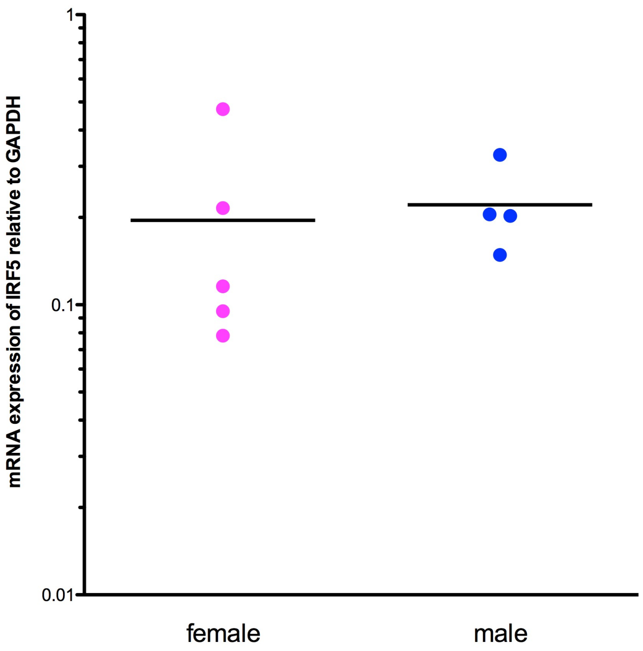

Figure S3: mRNA expression levels of IRF5 in isolated B cells.
mRNA expression levels of IRF5 in isolated B cells derived from females and males relative to GAPDH. No sex differences in expression levels of IRF5 mRNA could be detected (p=0.78, two-tailed t-test; females n=5, males n=4).

Antibody	Clone (Cat#)	Fluorophore	Manufacturer	Dilution	Application
anti-IRF5	E7F9W (76983)	unconjugated	Cell Signaling Technology	1:100	Flow cytometry
anti-CD3	UCHT1 (564307)	BUV737	BD Bioscience	1:100	Flow cytometry
anti-IgM	MHM-88 (314516)	BV421	Biolegend	1:100	Flow cytometry
anti-IgD	IA6-2 (348208)	Per-CP-cy5.5	Biolegend	1:100	Flow cytometry
anti-CD20	2H7 (302312)	PE-Cy7	Biolegend	1:40	Flow cytometry
anti-TNF-a	MAb11 (502936)	BV605	Biolegend	1:100	Flow cytometry

Table S1: Antibodies used in this study.
